# Rapid Detection of Nanoplastic Contamination in Plastic Labware by Dynamic Light Scattering Highlights Variations in Experimental Precision

**DOI:** 10.1021/acsmeasuresciau.5c00142

**Published:** 2026-01-07

**Authors:** Wei Wei, Song Lin Chua

**Affiliations:** † Department of Applied Biology and Chemical Technology, 26680 The Hong Kong Polytechnic University, Kowloon 999077, Hong Kong SAR, China; ‡ State Key Laboratory of Chemical Biology and Drug Discovery; Research Centre for Deep Space Explorations; Research Institute for Future Food, The Hong Kong Polytechnic University, Kowloon 999077, Hong Kong SAR, China

**Keywords:** dynamic light scattering, nanoplastics, microplastics, plastics, pollution, labware

## Abstract

Nanoplastics (NPs) are emerging contaminants of environmental concern, raising significant alarms due to their prevalence and potential health risks. Unlike larger microplastics, NPs are challenging to detect due to their nanodimensions and the reliance on labor-intensive methods such as nanoparticle tracking analysis (NTA) or scanning electron microscopy (SEM). This underscores the urgent need for rapid and accessible detection methods. To address these challenges, we employed dynamic light scattering (DLS), a widely used technique for measuring nanoparticle sizes, to rapidly quantify NP concentrations and sizes. Using DLS, we demonstrated the prevalence of NPs originating from laboratory-based plastic consumables such as microcentrifuge tubes, cryovials, and Petri dishes. Notably, routine actions, including pipet-tip scraping against plastic labware during sample handling, can introduce NPs into solutions. Moreover, physical or chemical procedures, especially sonication and liquid nitrogen treatment, further exacerbate the NP release. This interfered with experimental outcomes, including skewing of DNA and iron nanoparticle concentrations. Our material analysis revealed that the NPs were made of polystyrene and polypropylene, which correlated to manufacturers’ product details. Hence, our study highlights an under-recognized NP source that compromises research integrity while contributing to global NP pollution, thus emphasizing the need for sustainable laboratory practices and robust contamination control.

## Graphical abstract

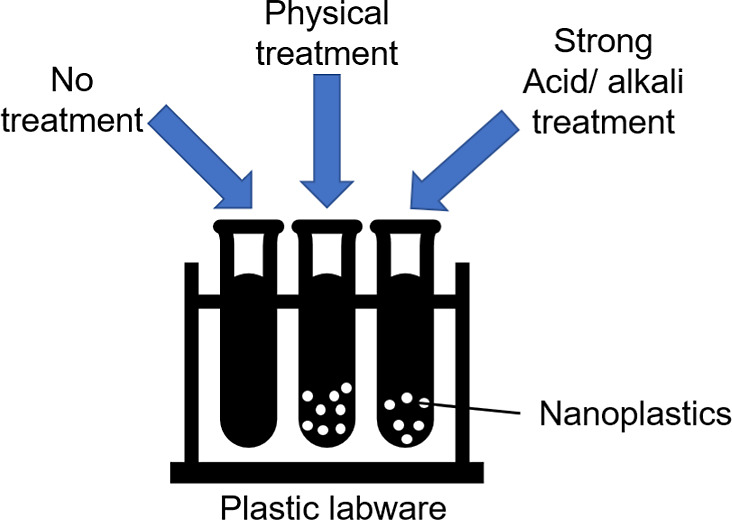

## Introduction

Plastic pollution has become a defining environmental issue of our time, with negative impacts on global ecosystems and human health.^1,2^ While much attention has been given to microplastics (MPs),^3,4^ nanoplastics (NPs, <1 μm) remain a relatively understudied contaminant of emerging concern. NPs pose potential risks to human health and wildlife,^5,6^ as their size enables them to penetrate biological tissues and accumulate in various environmental matrices better than MPs.^7,8^ An overlooked source of potential NP contamination lies in the ubiquitous use of plastic labware in scientific research, which raises the urgent need to evaluate the extent of NP contamination in research experiments. Since many research fields, such as nanotechnology and molecular biology, require precise measurements of samples, this raises the possibility that NP contamination could cause inaccurate measurements of nanomaterials or biological samples.

Despite these growing concerns, detecting and quantifying NPs remains a significant challenge. Conventional MP and NP detection methods, such as scanning electron microscopy (SEM), nanoparticle tracking analysis (NTA) and Raman microspectroscopy (μRaman), provide valuable insights into MP and NP morphology and composition but remain limited by high instrumentation costs, lengthy sample preparation, and low throughput, thus limiting their practicality for widespread monitoring of NP pollution in the environment.^9,10^ Specifically, NTA is widely used to estimate NP size distributions and concentrations in water samples,^11^ though its accuracy decreases at lower particle concentrations and with heterogeneous mixtures.^12^ Next, SEM provides detailed morphological visualization of NPs but requires complex sample preparation and vacuum conditions that may alter particle integrity.^13^ While μRaman offers chemical fingerprinting for polymer identification, it is time-consuming, has poor resolution to observe NPs, and is limited by signal interference from organic matter.^14^ Use of biosensors to detect MPs or NPs is also a viable option,^15^ but they may contaminate biological samples. In contrast, dynamic light scattering (DLS) has been employed to characterize nanoparticle size and concentration in colloidal suspensions, offering rapid measurements with minimal sample processing.^16^ Hence, DLS appears to be a practical and accessible approach to evaluating NP contamination.

Although leaching or shedding of MPs and NPs from commonly used household plastic containers has been reported,^17^ systematic quantification and real-time monitoring of NP contamination in laboratory settings remain underexplored. This gap is particularly concerning for research fields, such as nanotechnology, molecular biology, and analytical chemistry, where assays often rely on optical or surface-sensitive measurements that could be influenced by unintended NP interference. This raises the rationale to investigate whether plastic labware could serve as an overlooked source of NP contamination that potentially affects the experimental precision.

For rapid and accurate NP detection, we adapted the dynamic light scattering (DLS) technique, which is commonly used to measure size distribution and concentration of nanosize particles, ranging from metal nanoparticles to proteins and viruses.^18–20^ We showed prevalent NP contamination in plastic labware, which was exacerbated by physiochemical methods, resulting in the distorted measurements of biological samples and metal nanoparticles. First, measuring concentration and purity of DNA samples often requires the use of a NanoDrop spectrophotometer that relies on absorbance readings at specific wavelengths, typically 260 nm for DNA and 280 nm for protein.^21^ However, the presence of NPs could interfere with these readings, leading to different concentrations and reduced accuracy in purity assessments as NPs can scatter light and absorb at similar wavelengths.

Next, using iron nanoparticles as a proof of concept, we also showed that NP contamination could also distort the measurement of metal iron particles by DLS. While effective for nanoparticle quantification, DLS also yielded inaccurate results when NP contaminants are present, as NPs could alter sample composition and create misleading particle counts and size distributions. In summary, NP contamination could alter accurate measurements of both biological and nanomaterial samples, which warrants the need to improve experimental practices that reduce NP contamination.

## Materials and Methods

### Preparation of Nanoplastic Standards

NP standards were prepared from 100 nm commercial NPs (Kexinda, China) of different material types (polystyrene (PS), poly­(methyl methacrylate) (PMMA) and polypropylene (PP)). These polymer types of NPs were selected to represent the wide spectrum of polymer types commonly used in laboratory consumables, allowing us to assess DLS performance across different refractive indices and densities and ensuring the broader applicability of our method.

Milli-Q H_2_O (Millipore, USA) was added to NPs standards to various final concentrations (0, 1, 10, 100 ng/mL, and 1, 10, 100 μg/mL) in clean glass tubes. Subsequently, all standards were treated in an ultrasound sonicator (Elmasonic P120H, power = 50%, frequency = 37 kHz) on ice slurry for 30 min, followed by vigorous vortexing with a vortex mixer (Vortex Genie 2, USA) for 30 s to homogenize and avoid aggregation of NPs.

### NPs Contamination in Plastic Consumables

In order to better study NPs contamination in labware, frequently used plastic consumables in laboratory, including 1.5 mL microcentrifuge tubes (MCT), 50 mL centrifuge tubes (CT), 14 mL round-bottom test tubes, standard Petri dish (D35 mm), 96-microwell plates, and 2 mL cryovials from 3 different brands, were chosen. Due to legal and confidentiality reasons, the specific brands and manufacturers of the plastic labware could not be disclosed. According to manufacturer’s guide, the plastic labware was made from PS and PP.

All plastic labware was newly used without any prior treatment for testing, as received from the manufacturer to prevent preexisting contamination. To simulate scraping of pipet tips against container wells during introduction or extraction of liquids during experiments, pipet tips were used to scrape the walls of these chosen consumables randomly for 3 times, followed by the addition of 100 μL of Milli-Q H_2_O into different plastic consumables.

### Physical and Chemical Treatments of Plastic Consumables

To further evaluate NPs contamination after physical and chemical treatments, common treatments were conducted on pristine or scraped plastic labware as follows:

- Ultrasound sonication in water: Plastic labware were placed in ultrasound sonicator (Elmasonic P120H, power = 50%, frequency = 37 kHz) for 30 min.
- Heat treatment: Plastic labware were placed in a dry oven at 80 °C for 48 h.
- Autoclaving treatment: Only plastic labware which could be autoclaved (1.5 mL MCT) were autoclaved with pressurized saturated steam at 121 °C for 20 min at a pressure of 205 kPa.
- Freezing treatment: Plastic labware were placed in a refrigerator at −20 °C for 48 h, followed by thawing at room temperature for 1 h.
- Liquid N_2_ treatment: Plastic labware (1.5 mL MCT and 2 mL cryovials) were placed in liquid N_2_ for 24 h followed by thawing at room temperature for 1 h.
- Acid treatment: 1 mL of 1 M HNO_3_ (Sigma-Aldrich, Germany) was added into the plastic labware, followed by incubation at room temperature for 24 h.
- Alkali treatment: 1 mL of 1 M NaOH (Sigma-Aldrich, Germany) was added into the plastic labware, followed by incubation at room temperature for 24 h.

As negative controls, the plastic ware underwent identical handling except without physical or chemical treatments.

### NP Quantification Using Dynamic Light Scattering Technique

The DLS measurement was conducted by a DynaPro NanoStar (Wyatt, USA). Prior to sample introduction, the quartz cuvette was rinsed with Milli-Q water, followed by wiping with lint-free lens tissue dipped in HPLC-grade ethanol to ensure that the quartz cuvette surface is dust-free. 45 μL of samples from NPs standards (PS, PMMA, and PP) and plastic consumables were premixed in glassware to prevent secondary contamination from plastic labware and then carefully added into the quartz cuvette without touching its walls.

Prior to measurement, the DLS instrument was prewarmed for 30 min before placing the cuvette in the device where the temperature was maintained at 25 °C. After the temperature equilibrated in the instrument, the measurements would be performed. The DLS acquisition time was set as 5 s, with the number of DLS acquisitions set as 10. Each sample was measured 3 times to confirm the result. DYNAMICS software was used to control the instrument and collect the data. Based on the measured intensity, the NP concentration could be referred to by using the standard curves specific to various polymer types.

### NP and Nanoparticle Quantification Using Nanoparticle Tracking Analysis

To quantify NPs and iron nanoparticles, the NTA Instruments was used, as previously described.^9^ The 1 mL sample from NP standards (PS, 100 nm) at varying concentrations (0, 1, 10, 100 ng/mL, and 1, 10, 100 μg/mL) was introduced into the chamber of the NTA Instruments (NS 300 MALVERN, UK) and was recorded for 60 s. Every sample was run three times. A standard curve was established to determine the relationship between concentration and the number of tracked particles so as to quantify an unknown sample by measuring the NTA signal.

### Identification of NPs Using Fourier Transform Infrared Spectroscopy

FTIR (Thermo Scientific Nicolet IS50 FTIR Advanced XT-KBr Gold Spectrometer, USA) was used to characterize and identify the polymer composition and type. As previously described,^22^ all samples were transferred to and concentrated layer by layer onto aluminum foil. After drying for 24 h in an oven to remove moisture, the samples were mixed with KBr powder using the hydraulic press. Transmittance mode was applied for the FTIR spectroscopic analysis with 32 scans and the resolution of 4 cm^–1^ at the wavelength range of 4000–400 cm^–1^. The OMNIC software suite was used for advanced data collection and analysis.

### Morphological Characterization of NPs Using Transmission Electron Microscopy

The NPs were visualized by TEM (Thermo Fisher Talos L120C 120 kV TEM System equipped with a LaB6 filament and a Ceta 16 M CCD camera, USA). The 3.5 μL samples were transferred onto carbon film grids pretreated with a vacuum ion pump, left to stand for 30 s, followed by adding 3.5 μL of uranyl acetate for negative staining for 45 s. Excess liquid was wicked away with filter paper, and the grids were air-dried at room temperature. Triplicate images were captured, and representative images were presented.

### DNA and Protein Measurement Using NanoDrop

As previously described,^23^ *Pseudomonas aeruginosa* PAO1 culture was cultivated in 2 mL of LB media at 37 °C for 16 h, followed by washing of cells in the same volume of saline (0.9% w/v NaCl) and adjustment of the O600 to 1.0. Bacterial DNA extraction was performed by Nucleic Acid Kit (TianGen, China) per the manufacturer’s instructions, whereas protein extraction was performed using a sonifier (Branson, USA) and Tris–HCl (Sigma-Aldrich, Germany), according to the modified Pierre-Alain et al. protocol.^24^

Since DNA and protein samples were commonly placed and stored in 1.5 mL MCT, we compared DNA and protein samples placed in these containers and 1.5 mL glass tubes (plastic-negative control). NP standards (PS, 100 nm) were added into glass tubes at different concentrations (1, 10, 100, and 1000 μg/mL) to simulate NP contamination. Other plastic MCT and glass tubes were scraped by pipet tips randomly. Extracted DNA and protein were then introduced into these tubes for subsequent measurement by a NanoDrop One (Thermo Fisher, USA) with 2 μL of each mixed sample.

### Preparation of Iron Nanoparticles for DLS

Nanoparticles were prepared from commercial iron nanoparticles (Zhichuan, 100 nm, China). Milli-Q water was added to iron nanoparticles to 0.1 mg/mL in a clean glass tube and treated in an ultrasound sonicator for 30 min, followed by vigorous vortexing for 30 s. The iron nanoparticle suspension was added into the plastic labware for analysis by DLS.

### Statistical Analysis

All experiments were conducted in three independent biological replicates, each comprising technical triplicates to ensure reproducibility and accuracy. Data are presented as mean ± standard deviation (s.d.). Statistical analyses were performed using GraphPad Prism. One-way ANOVA was applied for comparisons among multiple groups, while paired Student’s *t* tests were used for comparisons between two related groups, as appropriate based on the experimental design. These tests were selected to assess statistical significance under assumptions of normality and homogeneity of variance, supported by the sample structure and replicate consistency.

## Results

### Detection of NPs Using DLS

We first validated the ability of DLS to measure both the size and concentration of NPs. First, we verified the detection sensitivity using standard nanoplastics, specifically commercially available polystyrene standards with specified sizes. We first showed that particle size measured by DLS was directly correlated to the known sizes (100 nm) of our NP standards (Figure 1A). The slightly larger measured size (101–150 nm) reflected the hydrodynamic diameters measured in DLS, which typically exceed nominal dry particle diameters due to surface hydration and possible mild aggregation.

**Figure 1:**
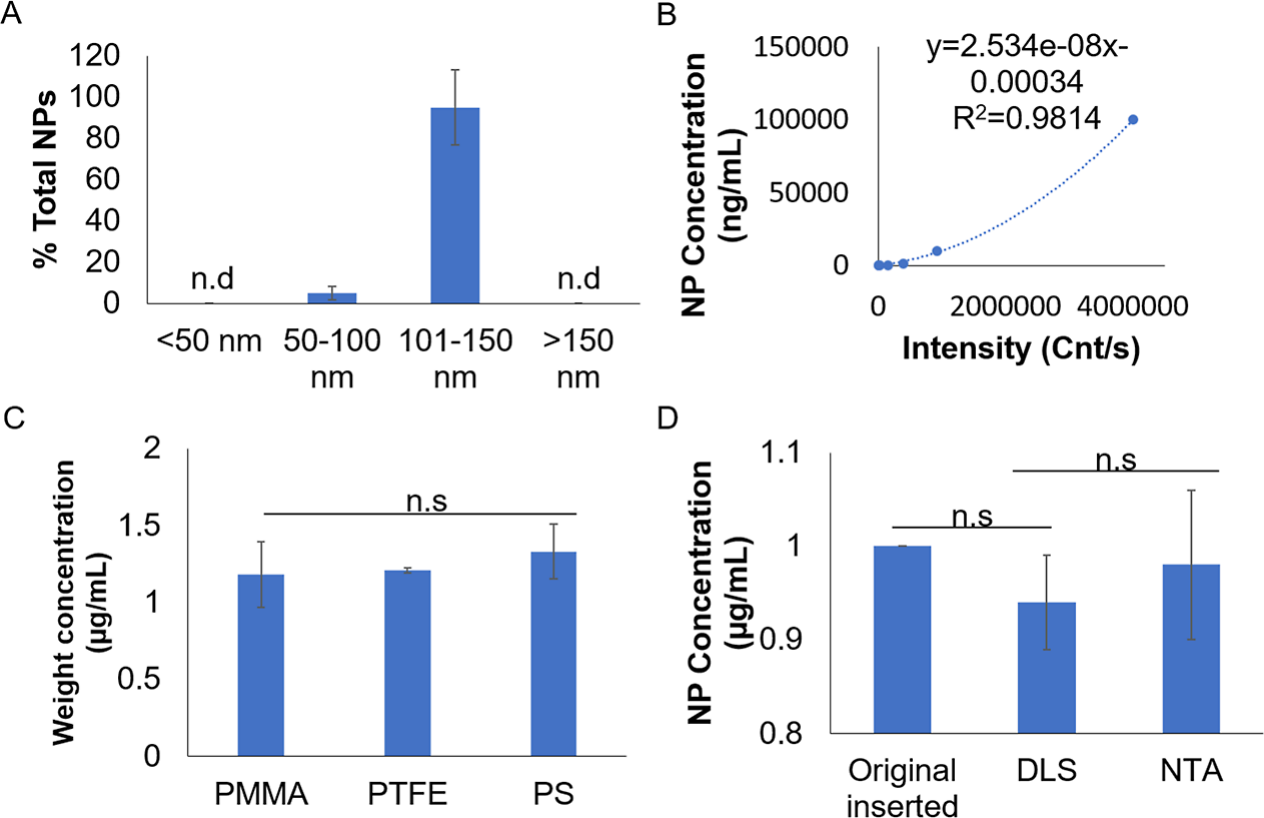
DLS detection of NP size and concentration. (A) Correlation of the DLS-detected size distribution to the standard sizes of PS particles. (B) Correlation of signal intensity to the concentration of standard PS particles. (C) Different material types of NPs do not affect DLS measurements. (D) No significant difference in using DLS and NTA to detect original 1 μg/mL NPs placed inserted into the tube. Mean ± sd. from three individual experiments are shown. N.s: not significant.

Other than knowing NP sizes, measuring NP concentration is a useful tool to evaluate severity of NP pollution in any environment. Using commercial PS nanospheres of varying concentrations, a linear relationship was observed between the signal intensity and concentration (Figure 1B and Supporting Information S1). This good correlation (*R* ^2^ = 0.994) provides powerful utility in estimating NP concentration, where [1] actual concentration of NPs could be estimated from the acquired standard curve and [2] the upper detection limit of DLS could be determined to reach a concentration of 100 μg/mL. We also observed similar linear standard curves for PMMA and PP standards (Supporting Information Figure S1 and Supporting Information S1). While DLS is unable to discern material types of NPs, different materials of NPs did not alter the accurate measurement by DLS (Figure 1C). This showed that DLS measurement would not be affected by different types of materials. To confirm that DLS is reliable, we performed the conventional NTA technique for comparison to DLS, and we showed that both methods could accurately detect NPs (Figure 1D).

### Presence of NPs in Plastic Consumables

Using DLS, we profiled NP contamination in various laboratory plastic labwares, including 1.5 mL MCT, 14 mL RBT, 50 mL CT, Petri dishes, 96-microwell plates (MWP), and 2 mL cryovials (CV) (Figure 2A). Surprisingly, even the simple scraping of pipet tips against the walls of plastic labware during the dispensing of liquid samples could introduce further NPs in plastic labware (Figure 2A).

**Figure 2:**
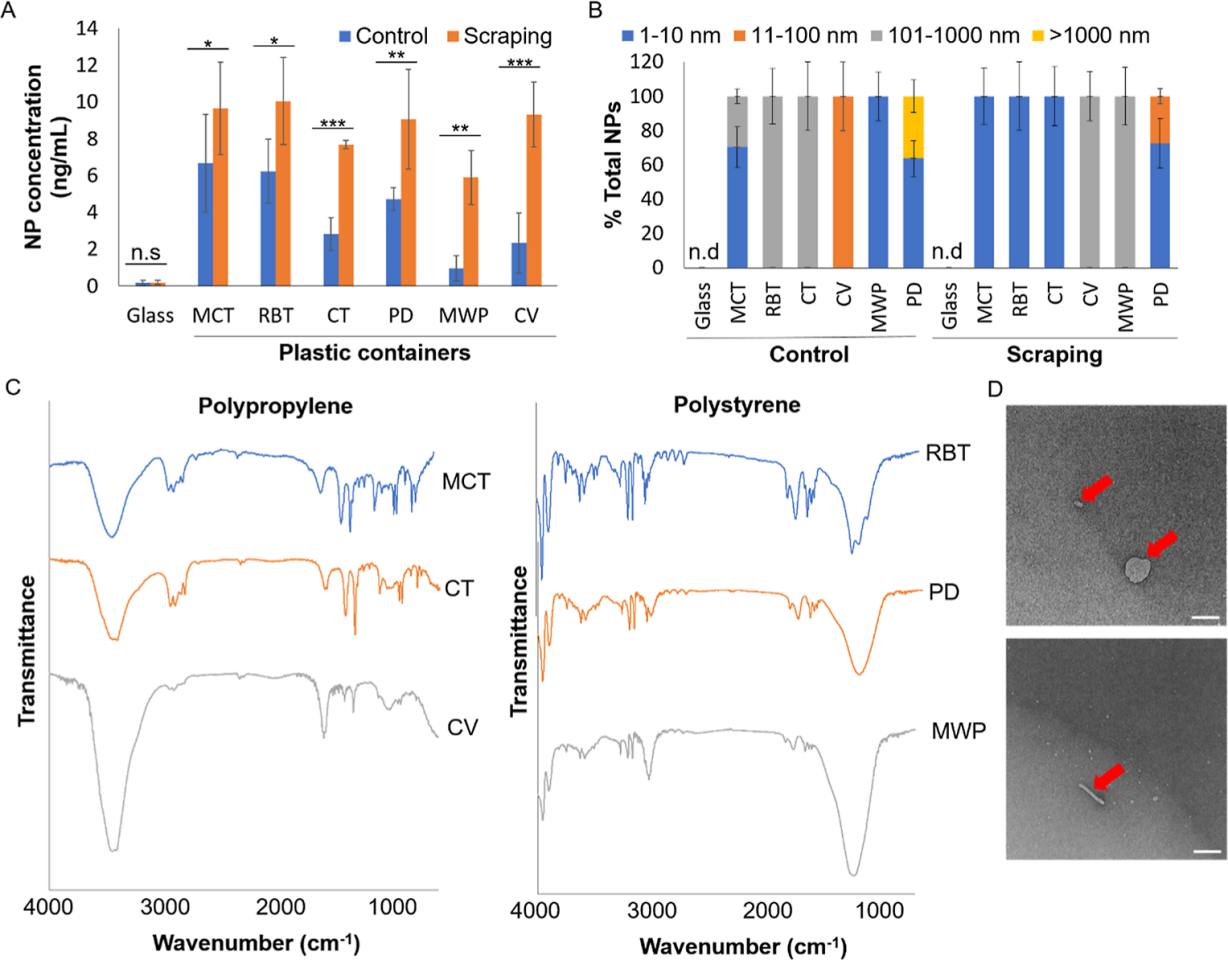
NPs were present in laboratory plastic consumables. (A) Concentration and (B) size distribution of NPs in different plastic consumables with and without pipet tip scraping. Mean ± sd. from three experiments are shown. N.s: not significant. ***P < 0.001, **P < 0.01, *P < 0.05. (C) Nano-FTIR spectrum of NPs released from plastic consumables. (D) NP appearances under TEM observation after scraping of the 2 mL microcentrifuge tubes with the pipet tip. Scale bar: 100 nm.

We next characterized the NP size, material, and morphology. The size of NPs was heterogeneous with a wide range from 1 to 1000 nm (Figure 2B), indicating that NPs were fragmented from the plastic containers. Next, the NPs were polypropylene (PP) or polystyrene (PS) particles (Figure 2C), which corresponded to the material type that was listed by the product manufacturers, indicating that the NPs originated directly from the labware. We then employed TEM to observe the morphologies of the NPs after scraping the MCT (Figure 2D) and other plastic labware (Supporting Information Figure S2), where NPs come in fragments with irregular shapes. This showed that NPs originate from fragmentation of the plastic tubes, including the mechanical scraping using pipet tips.

### Physicochemical Treatment Exacerbates NP Contamination

As research often involves various physical and chemical treatments of samples in their laboratory plastic containers, we next evaluated the effects of physical and chemical treatments on generating NPs. For physical treatments, we subject the plastic containers to sonication, heating (80 °C), autoclaving, freezing (−20 °C), or liquid N_2_ treatment (Figure 3A). As compared to nontreated control, all treatments could exacerbate NP contamination to varying degrees. Specifically, sonication and liquid N_2_ treatment generated the most NPs, possibly due to their harsh treatment on plastics. Furthermore, prior damage by pipet tip scraping to the walls of plastic containers could worsen the NP prevalence caused by sonication, or liquid N_2_ treatment was worsened by the presence of pipet tip scraping (Figure 3A), indicating that any predamage to the plastics could increase the susceptibility of plastics to physical damage. We also found that the sizes of NPs released after physical treatment of labware were highly varied (Supporting Information Figure S3).

**Figure 3:**
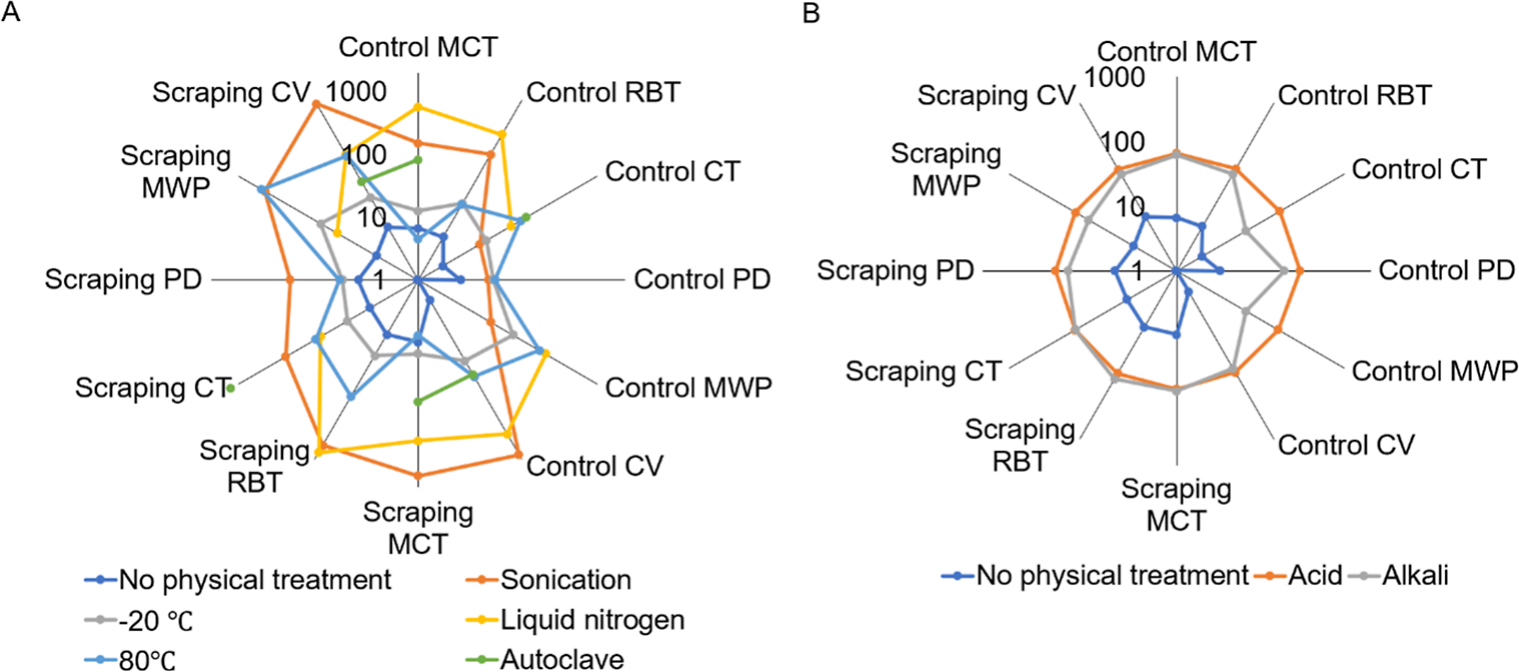
Physicochemical treatments exacerbated NP contamination in laboratory plastic consumables. (A) Concentration of NPs (μg/mL) released by physical treatments on plastics. (B) Concentration of NPs (μg/mL) released by chemical treatments on plastics.

Next, for chemical treatments, we subject the plastic containers to acidic (nitric oxide) or alkali (sodium hydroxide). Both acid and alkali treatment could generate NP, which could be worsened by the prescraping by pipet tips (Figure 3B). However, as compared to physical treatments, chemical treatments are considered milder in generating NPs, possibly because plastics are nonreactive to harsh chemical treatments in general. As for the size of NPs, acid treatment had the tendency to generate smaller NPs (up to 140 nm), whereas alkali treatment generated NPs of varied sizes (from ∼10 to ∼900 nm) (Supporting Information Figure S4).

### NP Contamination Alters Quantification of Iron Nanoparticles and Biological Samples

Since we had identified NPs in laboratory plastic consumables, this raises the possibility that NP contamination could affect the precise measurements of small samples. We found that the presence of NPs could alter measurements of iron nanoparticle concentration and size (Figure 4A,B). Moreover, NPs could affect the measurement of DNA by a common microvolume spectrophotometer (NanoDrop), where DNA concentrations in glass containers deviate from those in the plastic tube containing spiked NPs or the pipet tip-scraped tube (Figure 4C). The reduced DNA purity with NP contamination was reflected in the lower *A*_260_/*A*_280_ ratio (Figure 4D), where the standard ratio for pure DNA is ∼1.8. We also observed lower *A*_260_/*A*_230_ ratios in samples contaminated with high levels of NPs, where the standard ratio for pure DNA is ∼2.0, indicating “carbohydrate” contamination that directly correlated to the carbon-based NPs (Figure 4E). While low NP contamination (1 μg/mL spiked sample and scraped sample) might not reflect a major reduction in *A*_260_/*A*_230_ ratio (Figure 4E), it also indicated that NP contamination might go unnoticed.

**Figure 4:**
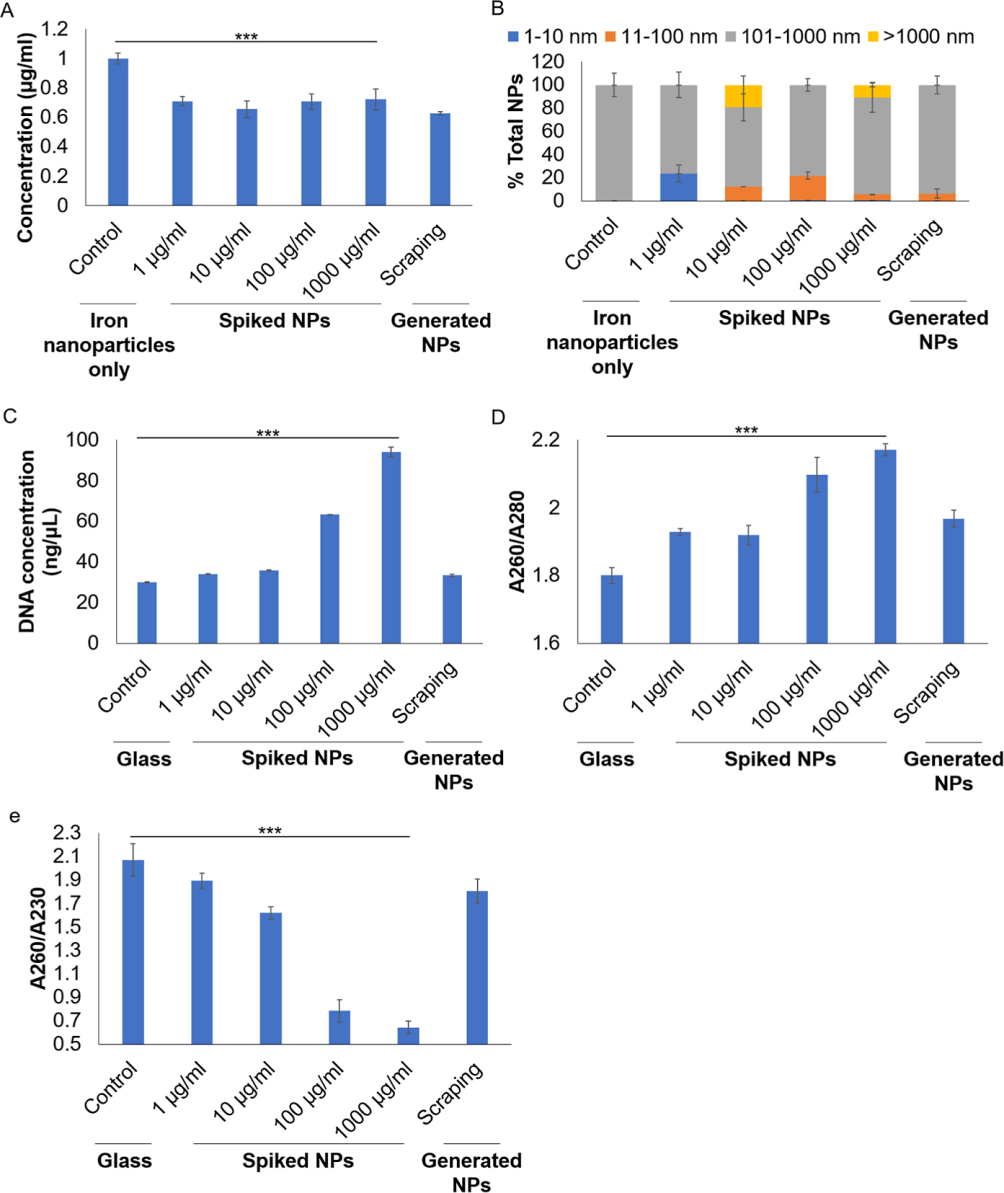
NP contamination alters the quantification of iron nanoparticles and biological samples. (A) Concentration and (B) size distribution of iron nanoparticles were altered by the presence of NPs. (C) Concentration of DNA was affected by NP presence. (D) *A*_260_/*A*_280_ ratio and (E) *A*_260_/*A*_230_ ratio of a DNA sample with and without NP contamination. Mean ± sd. from three individual experiments are shown. ***P < 0.001.

## Discussion

DLS is a common light scattering technique to measure the size distribution and concentration of nanosize particles in liquids, ranging from metal nanoparticles to proteins and viruses.^18–20^ We showed that DLS could be adapted for the nondestructive measurement of NP size and concentration in liquids, enabling the convenient, rapid, and cost-effective detection of NPs in large sample sizes. Given the difficulty to study NPs using laborious conventional techniques, DLS is a useful tool for rapidly monitoring NP pollution, especially NP size and concentration, as compared to other conventional physicochemical techniques (Table 1). Nonetheless, for a comprehensive NP analysis ranging from size to material type, multitechnique approaches are still required.

**Table 1:** Comparison of DLS to Other Conventional Techniques to Study NP Pollution

Technique	advantages	disadvantages
dynamic light scattering (DLS)	-rapid and user-friendly	-sensitive to polydispersity and aggregates
	-suitable for particle size range ∼1–1000 nm	-intensity-biased toward larger particles
	-nondestructive	-no information on chemical identity
	-minimal sample preparation	
	-cost-effective	
nanoparticle tracking analysis (NTA)	-measures individual particle size and concentration	-limited to ∼30–1000 nm
	-better resolution for polydisperse samples than DLS	-time-consuming
	-visual tracking of particles	-requires clean, dilute samples
		-no information on chemical identity
electron microscopy (EM)	-high-resolution morphological analysis	-requires vacuum and conductive coating
	-visual confirmation of particle shape and surface features	-no chemical identification without additional detectors, such as EDX
Raman microspectroscopy	-provides chemical composition	-limited sensitivity for particles <100 nm
	-enables polymer identification	-interferences from background
	-no staining or labeling required	-time-intensive
		-expensive instrumentation

However, DLS has several limitations to measure NPs.^25^ For example, it lacks the ability to discern material types, which requires nano-FTIR or Raman microspectroscopy to identify the types of NPs.^26–28^ Hence, these require a combination of different techniques used together for comprehensive separation, enrichment, and evaluation of NPs.^29^ Next, any artifacts, such as bubbles, can interfere with the accurate measurements by DLS, so a precise technique is required to prevent the introduction of bubbles. Lastly, it is highly sensitive to temperature changes and solvent viscosity, which warrants the need to maintain a constant temperature and determine solvent viscosity.

Next, our study reveals critical insights into the overlooked issue of NP contamination within laboratory environments by demonstrating that common laboratory procedures exacerbate the release of NP from plastic labwares. This could interfere with accurate measurements of experimental samples, leading to erroneous conclusions in research fields, requiring precise material characterization. Moreover, NP contamination may easily go undetected, leading to distorted experimental results and potentially compromising the reproducibility and integrity of the research findings. This highlights the urgent need to adopt stringent controls to monitor and minimize NP release during experiments.

To minimize NP contamination in laboratory experiments, we recommend several preventive measures based on our findings. First, glassware or metal alternatives that are inert and do not shed polymeric particles should be used whenever possible, especially for experiments involving nanoparticle analyses, nucleic acid quantification, or cell-based assays. Next, if using plastic consumables is unavoidable, prerinsing with ultrapure water or filtered buffer before use can effectively remove loosely bound surface NPs in the containers. Moreover, experimental procedures that involve intense mechanical or chemical stress should be minimized or optimized to reduce the level of polymer degradation and NP release. Lastly, we encourage the routine use of blank buffer-only controls to monitor potential background NP contamination. Implementing these practical strategies can help improve the reproducibility and reliability of nanoscale experiments by reducing unintended nanoplastic interference.

The findings of our study extend beyond the laboratory, revealing broader environmental implications that warrant our attention. NP contaminants from routine laboratory practices exemplify a pervasive and under-recognized source of contamination that can infiltrate ecosystems. The demonstrated release of NPs from plastic labware through physical and chemical treatments parallels the degradation processes occurring in natural environments, such as mechanical abrasion, thermal stress, and chemical exposure. This suggests that laboratory activities may inadvertently mimic and accelerate the mechanisms contributing to global NP pollution.

The persistence of NPs in the environment raises concerns due to their potential to accumulate and interact with various ecological systems. Once released, NPs can disperse into water, soil, and air, becoming vectors for transporting contaminants or interacting with biota.^26,30,31^ Their small size increases the likelihood of bioavailability, enabling NPs to penetrate biological barriers and accumulate within organisms, posing risks to food webs and ecosystem health. Furthermore, our findings underscore the need to reevaluate the environmental footprint of scientific research. Laboratories, often perceived as controlled and isolated environments, emerge as significant contributors to the growing challenge of plastic pollution, accounting for an estimated 5.5 million tons of plastic waste generated yearly.^32^ The ubiquity of disposable plastics in research settings, coupled with processes that exacerbate NP release, amplifies the potential for a long-term environmental impact.

The implications also extend to policy and industry. Regulatory frameworks that encourage the reduction of disposable plastics and promote the development of environmentally friendly materials in the laboratory are essential. Collaborations between researchers, policymakers, and manufacturers could drive innovation in sustainable materials and establish guidelines for minimizing environmental contamination from laboratory waste.^32,33^

In summary, our findings reveal the interconnectedness between laboratory practices and environmental health, highlighting the need for systemic changes to reduce NP pollution. By addressing this overlooked source of contamination, we can contribute to broader efforts to safeguard ecosystems and enhance the sustainability of scientific research.

## Supplementary Material





## Data Availability

Availability of data and materials: Data will be provided upon request from the authors.

## Notes

Standard curves of PMMA and PP NPs (Supplementary Figure 1), TEM observation of NPs (Supplementary Figure 2), and sizes of NPs in plastic labware after physical (Supplementary Figure 3) and chemical (Supplementary Figure 4) treatments (PDF)
(XLSX)
