# Supplementary material for: Rapid Detection of Nanoplastic Contamination in Plastic Labware by Dynamic Light Scattering Highlights Variations in Experimental Precision
Source: ACS Meas Sci Au. 2026 Jan 7;6(1):126–33. doi: 10.1021/acsmeasuresciau.5c00142 (PMC12921585; doi:10.1021/acsmeasuresciau.5c00142)
Supplement: Supplementary file 1 [file tg5c00142_si_001.pdf]

**Supporting Information**

# Rapid detection of nanoplastic contamination in plastic labware by dynamic light scattering highlights variations in experimental precision

**Authors and Affiliations:**

Wei Wei [1], Song Lin Chua [1, 2] *

1. Department of Applied Biology and Chemical Technology, The Hong Kong Polytechnic University, Kowloon, Hong Kong SAR China
2. State Key Laboratory of Chemical Biology and Drug Discovery; Research Centre for Deep Space Explorations; Research Institute for Future Food, The Hong Kong Polytechnic University, Kowloon, Hong Kong SAR China

*corresponding author:

Song Lin Chua

song-lin.chua@polyu.edu.hk

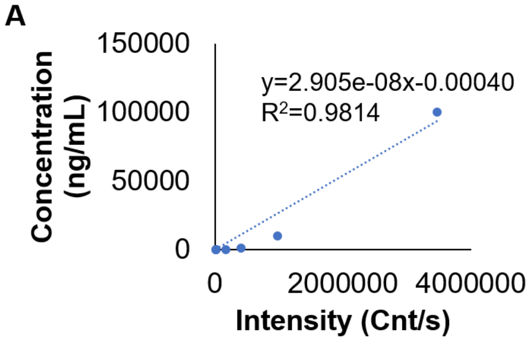


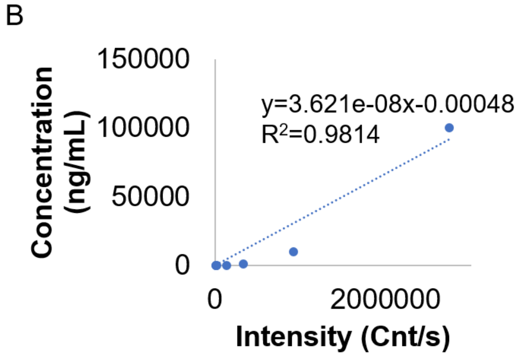


**Supplementary Figure 1.** Correlation of signal intensity to concentration of standard (A) PMMA and (B) PP nanoplastics.

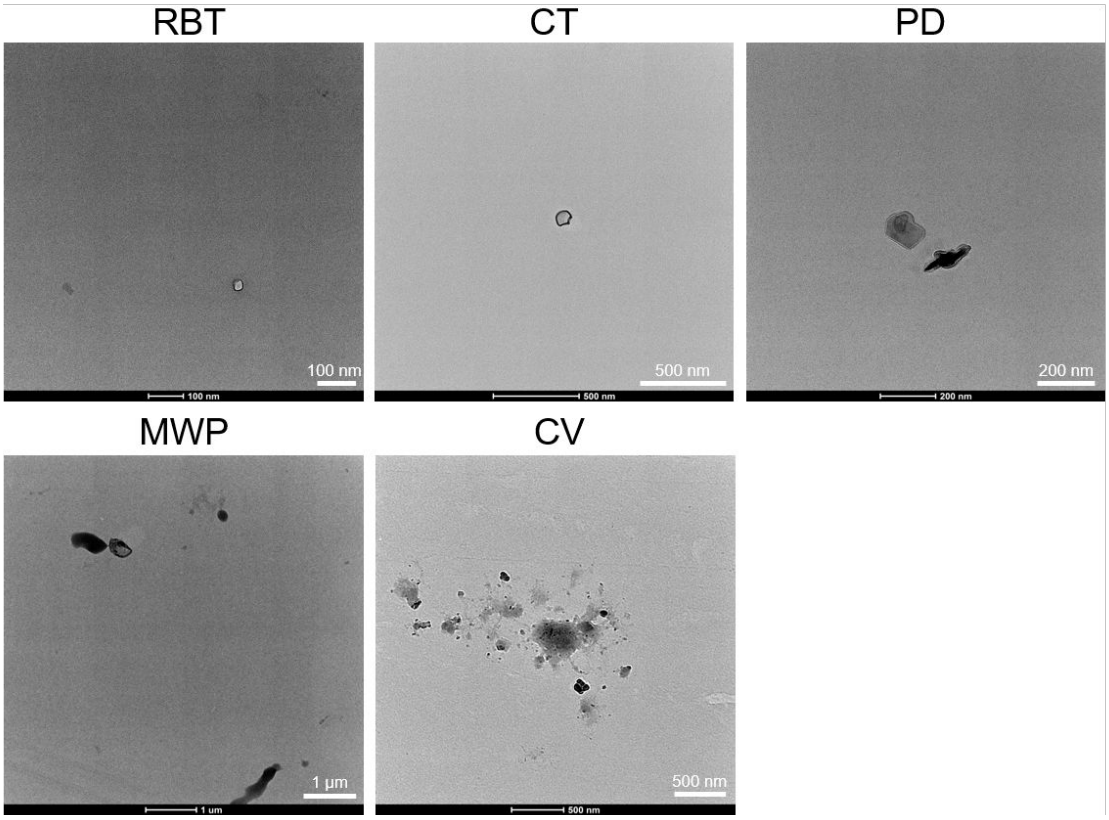


**Supplementary Figure 2.** Appearances of NPs released from 14-ml round-bottom test tubes (RBT), 50-ml centrifuge tubes (CT), petri dishes (PD), 96-microwell plates (MWP) and 2-ml cryovials (CV), under TEM observation.

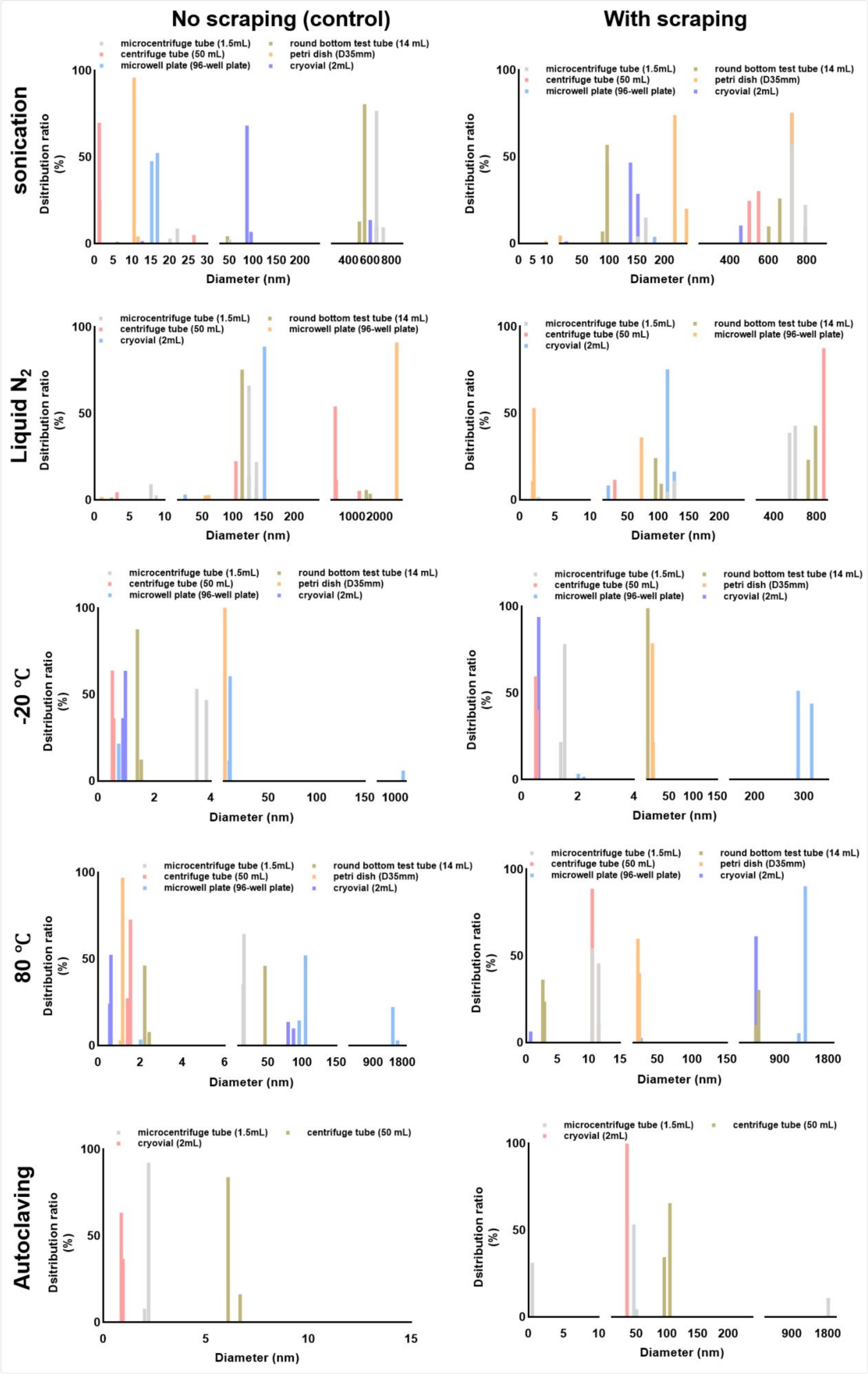


**Supplementary Figure 3.** NP sizes in plastic labware after different physical treatments, including heating (80 ºC), autoclaving, freezing (-20 ºC) or liquid $N_2$

treatment. Mean from three experiments are shown.

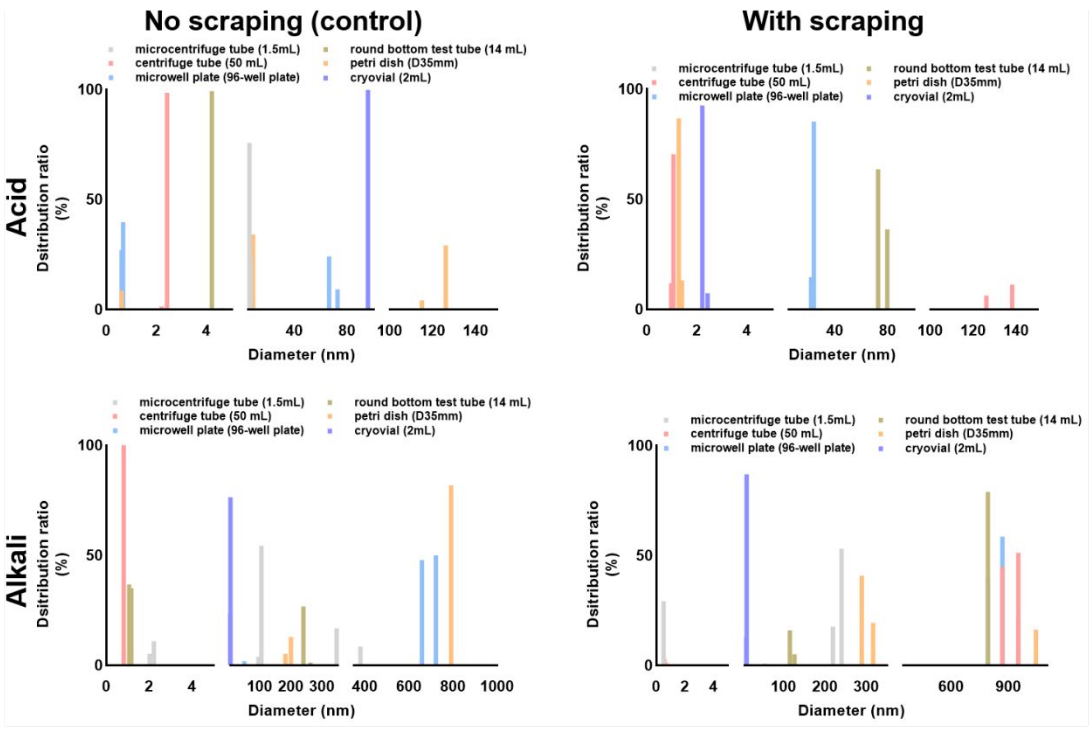


**Supplementary Figure 4.** NP sizes in plastic labware after different chemical treatments, including acid (nitric oxide) or alkali (sodium hydroxide). Mean from three experiments are shown.
